# Was There a Cambrian Explosion on Land? The Case of Arthropod Terrestrialization

**DOI:** 10.3390/biology11101516

**Published:** 2022-10-17

**Authors:** Erik Tihelka, Richard J. Howard, Chenyang Cai, Jesus Lozano-Fernandez

**Affiliations:** 1 School of Earth and Biological Sciences, University of Bristol, Bristol BS8 1TQ, UK; 2 Department of Earth Sciences, The Natural History Museum, London SW7 5BD, UK; 3 State Key Laboratory of Palaeobiology and Stratigraphy, Nanjing Institute of Geology and Palaeontology, and Center for Excellence in Life and Paleoenvironment, Chinese Academy of Sciences, Nanjing 210008, China; 4 Department of Genetics, Microbiology and Statistics & Biodiversity Research Institute (IRBio), University of Barcelona, 08028 Barcelona, Spain

**Keywords:** terrestrialization, artrhopods, Cambrian explosion, molecular clocks, palaeontology, phylogenomics

## Abstract

**Simple Summary:**

The transition of life from the aquatic realm onto land represented one of the fundamental episodes in the evolution of the Earth that laid down the foundations for modern ecosystems as we know them today. This key transition in the history of life is poorly known, owing to the scarcity of ancient terrestrial fossil deposits; complex terrestrial ecosystems with plants and animals appear in the fossil record during the Silurian and Devonian. However, recent molecular clock studies and new lines of palaeontological evidence point to a possibly much earlier origin of life on land, dating back as far as the Cambrian. Here, we review this controversy, using the arthropods as a case study of the possible cryptic Cambrian explosion on land. In particular, we highlight approaches for reconciling the disagreement between molecular clock estimates and the fossil record for the arthropod colonization of land.

**Abstract:**

Arthropods, the most diverse form of macroscopic life in the history of the Earth, originated in the sea. Since the early Cambrian, at least ~518 million years ago, these animals have dominated the oceans of the world. By the Silurian–Devonian, the fossil record attests to arthropods becoming the first animals to colonize land, However, a growing body of molecular dating and palaeontological evidence suggests that the three major terrestrial arthropod groups (myriapods, hexapods, and arachnids), as well as vascular plants, may have invaded land as early as the Cambrian–Ordovician. These dates precede the oldest fossil evidence of those groups and suggest an unrecorded continental “Cambrian explosion” a hundred million years prior to the formation of early complex terrestrial ecosystems in the Silurian–Devonian. We review the palaeontological, phylogenomic, and molecular clock evidence pertaining to the proposed Cambrian terrestrialization of the arthropods. We argue that despite the challenges posed by incomplete preservation and the scarcity of early Palaeozoic terrestrial deposits, the discrepancy between molecular clock estimates and the fossil record is narrower than is often claimed. We discuss strategies for closing the gap between molecular clock estimates and fossil data in the evolution of early ecosystems on land

## 1. Introduction

Molecular clocks estimate that life on Earth originated over 4 billion of years ago (Ga), perhaps shortly after the formation of our planet [1], with direct evidence provided by the remains of putative unicellular organisms at around 3.5 Ga (e.g., [2,3,4]). However, the emergence of complex multicellular organisms, such as animals, plants and fungi, only occurred during the last 1000 million years [5] (but see [6] for older estimates). The origin of animals gave rise to an enormous diversity of multicellular body plans, all with a complex embryonic development. This diversity of body plans is already seen in the exceptional early fossil record of animals, during the “Cambrian explosion”, beginning around 540 million years ago (Ma) and concluding perhaps as quickly as 521 Ma [7]. During this interval, most major animal phyla appeared almost simultaneously, from a geological perspective, and persisted throughout the Phanerozoic [8,9]). The often unfamiliar body plans of Cambrian marine animals have been preserved on a number of sites with exceptional preservation, known as the Burgess Shale-type (BST) *Konservat-Lagerstätten*, which provide a unique snapshot of the soft-bodied Cambrian biota in the sea [10]. A diverse and abundant marine arthropod fauna is evidenced by the fossil record from at least ~518 Ma, corresponding to the minimum age of the Chengjiang Biota of Yunnan Province, southwestern China; the oldest reliably dated BST [11].

Animals, plants, and life in general, have marine origins [12]. Only a handful of animal phyla contain lineages that can complete each phase of their life cycle outside of moisture-rich environments and can therefore be considered fully terrestrial. This is because land represents a new and hostile environment for marine organisms, with obstacles to overcome ranging from respiration, reproduction, feeding style, and mechanical support [13]. Among these, the most well-known examples are in vertebrates (reptiles, birds and mammals) and of course arthropods, invertebrates with jointed legs and exoskeletons such as spiders and insects. Additionally, soft-bodied groups with generally poor fossil records [14,15], such as molluscs (including the land snails and slugs [16]), onychophorans (velvet worms [17]), annelids (including earthworms [18]), nematoids (roundworms and horsehair worms, including many parasitic groups that have followed their hosts on land [18,19,20,21]), tardigrades (water bears [22]), and platyhelminthes (flatworms [23]) contain land-living lineages, but these are mostly dependent on moisture-rich terrestrial environments for survival. Life on land requires a series of adaptations that may be paralleled across different groups—we can refer to this as terrestrialization: the process by which aquatic organisms adapt to terrestrial life. Terrestrialization is a fascinating field of study in evolutionary biology. Much literature has addressed terrestrialization at the physiological level in arthropods (see review in [24,25]). However, most studies have been conducted on isolated lineages and have not taken full advantage of the comparative approach between diverse terrestrial groups [26]. Multiple and independent terrestrialization events allow comparisons of alternative solutions taken up by different groups to the same adaptive challenge, and represent a powerful tool to understand adaptation in an evolutionary framework. This information is, at the same time, necessary to be able to carry out comparative analyses and estimate the timing and rate of emergence of terrestrial adaptations. Although animal phylogenetic diversity (understood as the diversity of body plans) may be higher in the marine realm, terrestrial biodiversity is clearly higher in terms of the number of species—particularly due to the unparalleled species richness of insects [27]. Understanding animal terrestrialization is thus crucial to understanding the origins of biodiversity on Earth and the mechanisms underpinning evolutionary adaptation [28].

There is fossil evidence of simple terrestrial ecosystems formed by single-cell organisms dating back 1000 Ma [29]). The earliest complex terrestrial ecosystems record a fascinating transition in the history of life. Before the Palaeozoic, the only terrestrial life was unicellular, which, until recently, could only be deduced from indirect evidence [30]. It was during the Palaeozoic that plants and animals began to colonize the Earth’s landmasess [31], with plants appearing in the fossil record in the form of microfossils called cryptospores in the Middle Ordovician, around 470 Ma, with potential vascular land plants appearing shortly at ~458 Ma [32]. In the case of arthropods, with certain terrestrial myriapods and arachnids from the Silurian–Devonian [33,34]. Hence, the conventional view of the evolution of terrestrial ecosystems posits that during the Silurian–Devonian, animals and plants diversified on land, which was presumably void of complex organisms, bathed in lethal UV rays, and with low atmospheric oxygen (e.g., [35,36]). This model has however recently been challenged by molecular clock dating studies [25,37,38] and new discoveries of Palaeozoic stem groups of terrestrial lineage [39,40], which imply a substantially earlier, Cambrian to Ordovician, origin of complex terrestrial ecosystems, comparable to a “Cambrian explosion on land”. Secondly, updated reconstructions of Devonian-Carboniferous atmospheric oxygen suggest that this period did not suffer from substantially low atmospheric oxygen as stipulated earlier [41,42]. Meanwhile, terrestrial sedimentary rock units older than the Early Devonian are rare worldwide (e.g., [43,44,45,46,47]). For example, Western Europe, one of the best explored regions of the world from a palaeontological point of view, has virtually no terrestrial sedimentary rock outcrops older than the latest Silurian [15,48,49]. The scarcity of preserved rock units imposes an important constraint on the preservation potential of the earliest terrestrial ecosystems. It has been argued that the scarcity of terrestrial organisms from this period may be due to limited surviving fossiliferous sediments rather than because they did not exist in the first place [42,50]. This emerging paradigm may imply up to 100 million years of discordance between when diverse terrestrial ecosystems become represented in the fossil record and their putative origin.

**Figure 1:**
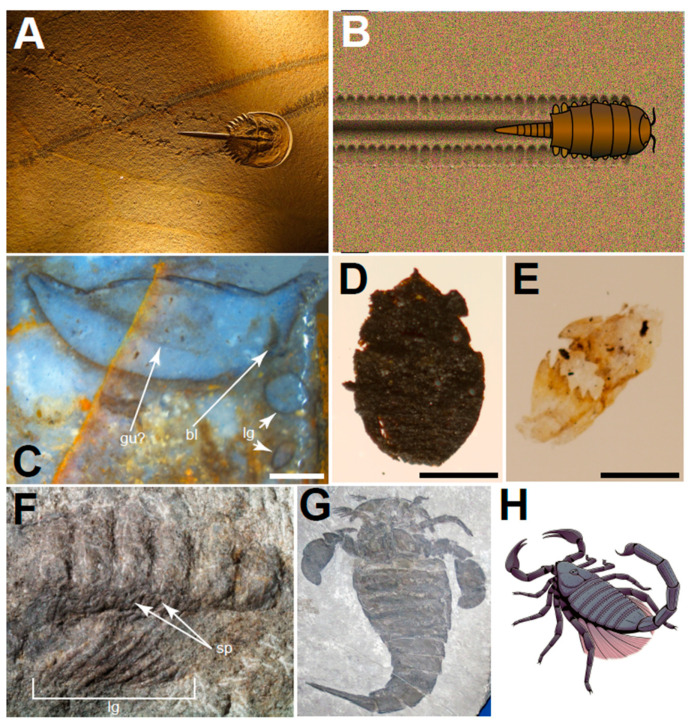
Fossil evidence of arthropod terrestrialization. (**A**) Traces and the body fossil of the horseshoe crab that made it, *Mesolimulus walchi*, morphologically resembling modern forms; (**B**) reconstruction of a terrestrial Cambrian ichnofossil, possibly made by the euthycarcinoid *Mosineia,* a group in kinship with myriapods; (**C**) Section through the abdomen of a trigonotarbid arachnid preserved in the Early Devonian Rhynie chert, revealing book lungs (bl), a possible trace of the gut (gu?), and sections through the legs (lg); (**D**) Carbonised body fossil of a trigonotarbid arachnid *Palaeotarbus jerami* from the Silurian Ludford Lane; (**E**) Putative myriapod mandibles from the Silurian Ludford Lane; (**F**) Millipede *Pneumodesmus newmani* from the Lower Devonian of Cowie Harbour (Scotland), presenting spiracles (sp) and legs (lg); (**G**) Eurypterid *Eurypterus remipes* from the Silurian; (**H**) Palaeoreconstruction of the Devonian scorpion *Waeringoscorpio westerwaldensis*, with filamentous gills that suggest a potential aquatic adaptation. Image sources: Wikimedia Commons Illustration authors: (**B**) Haug; (**C**–**E**), Erik Tihelka; (**H**) Junnn11 (@ni075). Institutional repositories: (**C**–**F**) National Museum of Scotland, Edinburgh: R.08.14 & G.2001.109.1; (**D**,**E**) Ulster Museum, Belfast: K25850 & LL1.6-23; (**G**) Generaldirektion Kulturelles Erbe, Direktion Archiologie/Erdgeschichte, Mainz, Germany, based on PWL2007/5000-LS. Scale bars: (**C**,**D**) 500 μm, (**E**) 250 μm, (**G**) ~10 mm.

In this brief review, we introduce the timescale of arthropod terrestrialization. Arthropods are represented among the oldest fossil records of animals (Figure 1), and represent the bulk of animal diversity on land today, with more than a million described species [51]. The oldest arthropod fossils are undoubtedly marine. They include the trilobites, with representatives dating back to the early Cambrian, ~521 Ma [52], and trace fossils indicating the presence of arthropod locomotion from at least ~528 Ma [53]. In arthropods, there have been a minimum of three to four major terrestrial invasions during the Palaeozoic: that of hexapods (which includes insects and kin), isopods (a group of crustaceans), myriapods, and that of arachnids—assuming that the latter forms a monophyletic group. The multiple and independent terrestrializations in arthropods provide a unique macroevolutionary case study into adaptative solutions embraced by different groups in response to the same challenge. More broadly, the topic of animal and plant terrestrialization provides an exciting opportunity to study a crucial ecosystem-wide transition that shaped the world we find so familiar today, during an elusive epoch of Earth’s history that left little direct physical evidence. However, to carry out these studies it is necessary to: (i) clarify how many land settlements have occurred independently in different arthropod lineages, (ii) estimate when these terrestrialization processes occurred and how long they lasted, and (iii) establish robustly which is the aquatic sister group of each terrestrial lineage. We provide an overview of recent progress in these questions and evaluate the support for the argument of a Cambrian explosion on land.

## 2. Origin and Terrestrialization of Arthropods 

### 2.1. Arthropod Origins

**Figure 2:**
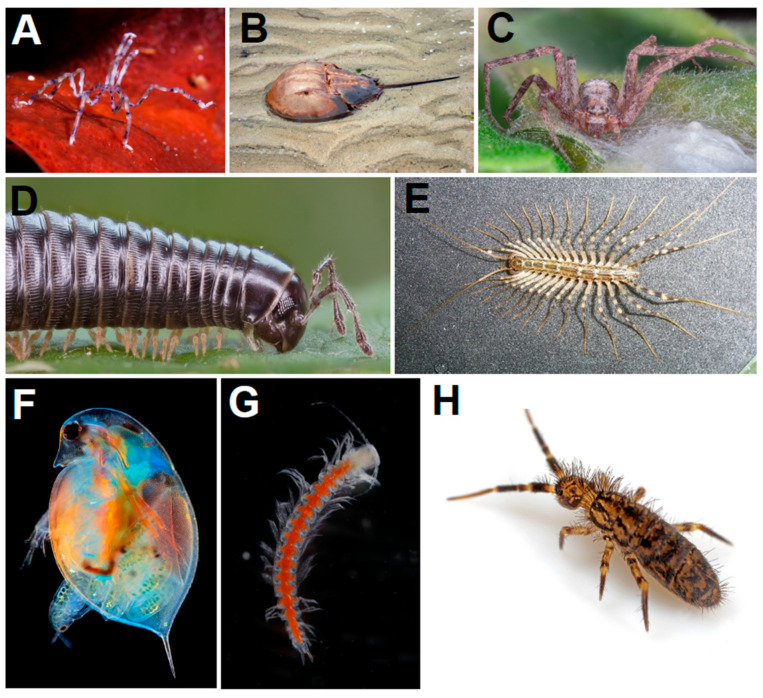
Present diversity of arthropods (**A**) pycnogonid *Endeis flaccida* (chelicerate); (**B**) xiphosuran *Limulus polyphemus* (chelicerate); (**C**) spider *Philodromus aureolus* (arachnid: chelicerate); (**D**) millipede *Cylindroiulus caeruleocinctus* (myriapod); (**E**) centipede *Scutigera coleoptrata* (myriapod); (**F**) branchiopod *Daphnia* sp. (pancrustacean); (**G**) remipede *Morlockia williamsi* (pancrustacean); (**H**) hexapod *Orchesella villosa* (pancrustacean). Image sources: Wikimedia Commons; (**G**) Jørgen Olesen.

It is difficult to precisely estimate terrestrial arthropod biodiversity in deep time due to the caveats of the fossil record; terrestrial arthropod fossils are usually limited to sites of exceptional preservation known as *Konservat*-*Lagerstätten*, and therefore their stratigraphic and environmental distribution is discontinuous. However, we can suppose that, as in the modern biosphere, arthropods were probably the largest component of the diversity and abundance of Palaeozoic land animals, given the lack of initial competition and the phylogenetic diversity of those that are present in the terrestrial Palaeozoic fossil record. Indeed, arthropods are likely to have been the dominant animal group in terms of biodiversity in perpetuity for the past 520 million years [54]. Arthropods are characterised by presenting internal and external body segmentation with regional specialisations (tagmosis: in the case of insects, for example, they possess a thorax where legs and wings are inserted while there are no extremities in the abdomen); an external skeleton composed of articulated sclerotized parts; body segments that originally had associated articulated limbs; growth through successive moults (ecdysis); and an open circulatory system with a dorsal heart with lateral valves [55]. This set of unique characteristics suggests that they are a monophyletic group (descendants of a common ancestor who possessed the diagnostic characteristics of the lineage). Arthropods are represented by chelicerates (with arachnids such as spiders and scorpions, and marine groups such as pycnogonids and horseshoe crabs); myriapods (such as millipedes and centipedes); hexapods (containing insects) and predominantly aquatic ‘crustaceans’ (for example crabs and prawns), which are collectively known as pancrustaceans; and include important extinct groups, such as the trilobites (Figure 2). Their abundance makes arthropods ecologically essential; for example, myriapods are important processors of detritus in forests, and termites consume such large amounts of cellulose that they are significant for the carbon cycle and atmospheric gas composition [56]. Without arthropods, life and ecosystems on Earth would be radically different. Their surprising diversity (which exceeds 75% of all living species described [57]) can help to elucidate the patterns and processes of macroevolution.

The earliest animals we know as land-dwelling were arthropods [58]. Evaluating the earliest fossil evidence of arthropod life on land can rely on two approaches—phylogenetic bracketing and direct anatomical evidence. Under the former approach, the discovery of a fossil representative belonging to an entirely terrestrial clade can be deemed to provide evidence of life on land, even when the state of preservation of the individual fossils is not particularly impressive. The second, more direct approach, relies on identifying unambiguous terrestrial adaptations in fossil specimens to conclude that these indeed lived on land. 

The earliest fossil assemblage preserving arthropods belonging to terrestrial clades is the Přídolí-aged Ludlow bone bed Member exposed at Ludford Lane, near Ludlow in Shropshire, western England [34,59,60,61]. This site contains a range of myriapods (Figure 1E), including scutigeromorph centipedes in the genus *Crussolume* [61], the arthropleurid *Eoarthropleura* [61], and a singular specimen of the trigonotarbid arachnid *Eotarbus jerami* Dunlop 1996 (= *Palaeotarbus jerami*, junior synonymy resolved by Dunlop [62]; Figure 1D). Any of these can be confidently considered to be the oldest terrestrial arthropod body fossils, albeit the fidelity of their preservation does not permit the observation of anatomical adaptations for life on land—most are represented by small shreds of cuticle or, in the case of *Eotarbus*, a dark carbonised specimen. U-Pb zircon dating of the Ludlow bone bed at Ludford Lane in Shropshire constrained the age of the deposit to ~420 Ma [63].

The earliest animal possessing unambiguous terrestrial adaptations is the millipede *Pneumodesmus newmani* from the Lower Devonian Cowie Harbour near Stonehaven in Aberdeenshire, Scotland [33], which is preserved with more fidelity. The terrestrial character of this organism is indisputable since it possesses spiracles, openings on the cuticle that allow air to enter the tracheal system (Figure 1F). Two other diplopod species were reported from the locality, all described by Wilson and Anderson [33]. The *Dictyocaris* Member of the Cowie Formation at Cowie Harbour was initially considered to be Silurian based on palynological evidence (~426.9 Ma [64,65,66]), but isotopic dating confidently constrained its age to the lowermost Devonian (Lochkovian; ~414 Ma [67]), making it some 6 Mya younger than the Ludford Lane assemblage. Recently, the scorpion *Palaeoscorpius devonicus* [68,69] from the Lower Devonian Hunsrück Slate Lagerstätte in Germany (~405 Ma) was interpreted as possessing adaptations for life on land, namely probable book lungs, indicating that it was likely terrestrial [70].

### 2.2. Arthropod Phylogeny

The evolutionary relationships among the major arthropod groups have always been a subject of debate, such that by the start of the 21st century virtually all conceivable topologies for the group had been proposed [71]. Identifying the closest relatives of each terrestrial lineage is crucial, not only for comparative studies dealing with adaptation strategies for life on land, but also to understand the potential terrestrialization routes and constrain their timing. To infer these phylogenies, the anatomical structures of living and fossil species provide a treasure trove of comparative data that has been expanded even further during the last few decades by vast quantities of molecular data [72]. In their adaptation to land, arthropods have undergone convergent evolution (independent origins of similar biological systems in different lineages), which has often complicated efforts to assess kinship relationships between them [54]. For example, trachea (respiratory structures adapted to terrestrial environments) are found in several lineages that have conquered the land independently during the Palaeozoic: in a few arachnids, myriapods, isopods, and hexapods. The introduction of genome-scale phylogenetic analyses-phylogenomics—has greatly narrowed down the number of hypotheses on hexapod phylogeny, but crucially, some nodes of the arthropod tree remain difficult to resolve. Such challenging nodes often represent ancient and rapid radiations that are complex to address with any dataset, molecular or morphological, and represent the major lasting controversies in reconstructing the process of the arthropod invasion of land [18,66,73,74,75].

### 2.3. Myriapods

**Figure 3:**
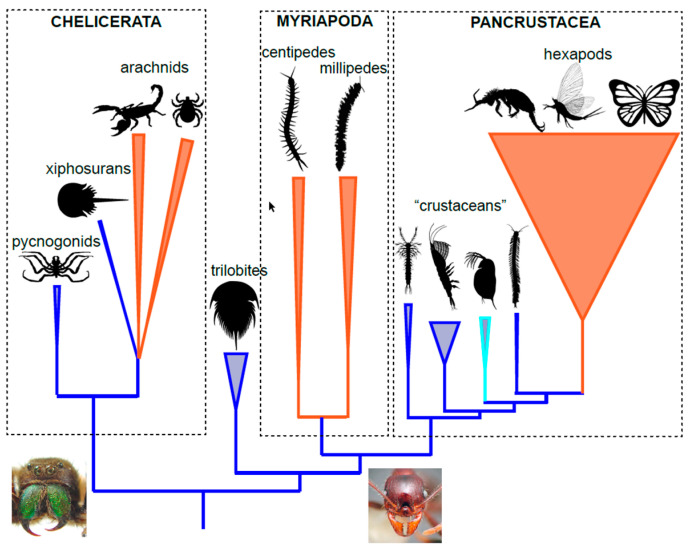
Cladogram with the current consensus on the phylogenetic relationships between the main groups of arthropods. The terrestrial groups are represented in orange colours while the marine clades in blue and turquoise for Branchiopoda (fresh water). The thickness of the terminal branches corresponds to a proportional approximation of the number of described species. At the base of the cladogram, image with detail of chelicerae and mandibles, the defining structures of the two groups. Some of the silhouettes come from Phylopic (phylopic.org/; accessed on 5 November 2022).

According to a classical phylogenetic hypothesis, the exclusively terrestrial myriapods, have been regarded as the sister group of the hexapods. This hypothetical clade, called Tracheata (or Atelocerata), is supported mainly by the presence of tracheae in both groups to carry out gas exchange (reviewed in [76]). Current studies based on molecular data, and also a re-examination of more subtle morphological characters of the nervous system and ommatidia [73,77], discard this hypothesis, and attribute this coincidental morphological convergence to independent convergence [78]. A second hypothesis recovered by early analyses of molecular data implicated myriapods as a sister group to the chelicerates (Myriochelata or Paradoxopoda). However, these results are now considered as caused by a phylogenetic reconstruction bias due to the rapid evolutionary rates of pancrustaceans attracting to the outgroup and pushing myriapods and chelicerates into an artefactual clade when using simpler models of molecular evolution [79]. Today, there is a certain consensus on the main relationships between arthropods, supported by phylogenomic data [78]. The myriapods, the first of the three large terrestrial lineages, are generally accepted as a sister group to the pancrustaceans (hexapods and all crustacean lineages), and the chelicerates as the closest relative of this clade (Figure 3). Thus, the basic division between arthropods consists of those that have mandibles (myriapods and pancrustaceans) and chelicerae. The internal phylogeny of myriapods, though, is currently more contentious. Several recent phylotranscriptomic analyses disagree on the exact relationship between their main lineages [78,80,81,82,83] but they do not have an impact on the single terrestrialization event inferred for the group.

### 2.4. Pancrustacea (Hexapoda)

There is strong molecular and morphological evidence that favours the position of hexapods as nested within the ‘crustaceans’ (as the clade Pancrustacea, or Tetraconata), and myriapods as the sister group of pancrusteans forming the Mandibulata group, characterized by the presence of this distinctive oral structure [84,85,86]. In contrast, the exact relationships of hexapods within the Pancrustacea are still unclear, and it is not obvious which is their aquatic sister group. Phylogenomic datasets have variously lent support to the mostly freshwater-dwelling branchiopods [25], or the species-poor and enigmatic remipedes [68,70]. Establishing which ‘crustacean’ group is the most closely related to hexapods has a great impact on whether the latter group presumably colonised terrestrial environments directly from the sea, or whether they first colonized freshwater environments and later moved to land. Most recent phylogenomic studies, though, using hundreds of molecular markers, have shifted the balance in favour of Remipedia [84,86]. Remipedia are a class of blind and predatory crustaceans that live in coastal aquifers that contain saline groundwater. They were discovered less than 40 years ago [87], and have a very restricted distribution, with fewer than 30 known species described from the anchialine caves in the Caribbean Sea, two species from the Canary Islands and one from Western Australia. Very little is known about the biology of these organisms, which makes it difficult to understand their significance for hexapod terrestrialization.

### 2.5. Pancrustacea (Isopods)

The suborder Oniscidea (woodlice) represents the most diverse isopod crustacean group, with over 3700 described species [88]. It is the only pancrustacean group besides the hexapods composed almost entirely of terrestrial species; its members are found in almost all terrestrial habitats, ranging from nearshore settings to forests [89]. In particular, the intertidal genus *Ligia* inhabiting shorelines is often regarded as a transitory group [90]. Given their varying degrees of adaptations for life in semi-aquatic and terrestrial environments, woodlice provide a rewarding model group for understanding the transition from marine to terrestrial habitats, which hinges on an understanding of their phylogeny [90,91]. Morphological studies implicate Ligiidae as the basalmost woodlouse clade, implying a single invasion of land directly from the marine realm [92], although some molecular studies have challenged the monophyly of the group (e.g., [93,94]). Overall, isopods remain probably the least-studied terrestrialization event among arthropods. Their fossil record is fragmentary and scarce, with their oldest occurrence from the Cretaceous (summarised in [90]). If terrestrial isopods originated in the late Palaeozoic, potentially the Carboniferous [90], they would represent the most recent arthropod terrestrialization event. 

In some sense, other pancrustacean clades such as amphipods and the decapods, also invaded semi-terrestrial habitats (e.g., supralittoral zone of beaches, most soil and leaf litter, edges of freshwater habitats) and these have been considered as terrestrialization events by some (e.g., [13]). Here, we refrain from treating these groups as fully terrestrial, since their adaptation to life is not as developed as in the case of the woodlice. Nonetheless, these taxa represent important study groups for future research in arthropod adaptation to semi-terrestrial habitats.

### 2.6. Arachnids

Among terrestrial arthropods, only insects outnumber arachnids in terms of the number of described species (1 million versus 112,000, respectively; [51]). The clade Arachnida includes all terrestrial chelicerates, composed mainly of predatory groups such as spiders and scorpions, and parasites such as ticks. However, chelicerates also include marine taxa such as the pycnogonids (sea spiders) and xiphosurans (horseshoe crabs). Neither the currently available morphological nor molecular data have unequivocally resolved the internal kinship relationships between chelicerates [66]. Arachnids have traditionally been regarded as a monophyletic group, implying that a single and irreversible ancestral colonization of land paved the way to this group’s evolutionary success. Some recent studies including genome-scale and morphological phylogenies, however, do not support this relationship, instead placing the marine Xiphosura within terrestrial arachnids, and not as a sister group to it [74,95]. The focus of this debate is whether there has been a single common ancestor for all terrestrial arachnids, a single terrestrialization event within a common ancestor of terrestrial arachnids + xiphosurans (with the later transitioning again into aquatic environments soon after), or whether arachnid terrestrialization occurred on two or more separate occasions. Resolving this puzzle is enormously significant, as it rewrites our perception of the evolution of terrestrial adaptations (e.g., the respiratory system, sensory and reproductive systems, and the locomotor appendages). The physiological demands of life on land require a significant modification of these anatomical features, which is probably best illustrated by the respiratory organs, a great variety of which are present in extant chelicerates (book lungs and tracheae in terrestrial groups, and book gills in marine forms) [96,97]. If xiphosurans were a group of marine arachnids, this may suggest that the remaining lineages colonized land independently. A second option would be that xiphosurans recolonized the marine environment from a terrestrial ancestor. Of these two options, the first would be considered more plausible, since the fossil record of Xiphosura extends back more than 400 Ma with exclusively aquatic forms, without traces of a potential terrestrial or amphibious ancestors [98] (Figure 1D). Furthermore, no widespread losses of terrestrial respiratory organs in arthropods are known, once acquired, in line with the predictions of Dollo’s law [99].

In addition, even though horseshoe crabs can make momentary incursions into the coasts to spawn eggs, they do not have distinctly terrestrial morphological adaptations and their body structures present great similarity, and probably homology, with that of other aquatic fossil chelicerates [100,101]. Other recent studies using genome-scale datasets, as well as morphological and fossil evidence suggest that marine chelicerates (pycnogonids and Xiphosura) are successive sister groups of a monophyletic lineage of terrestrial arachnids. These results are compatible with a single colonization of land within chelicerates and the absence of wholly marine arachnid orders [66,102].

## 3. Pre-Devonian Fossil Record of Terrestrial Arthropods

### 3.1. Trace Fossil Evidence

The oldest traces of activity in the terrestrial environment made by arthropods (ichnofossils) date from the middle Cambrian to the Early Ordovician. The oldest of these include trackways on land from the late Cambrian (~500 Ma) of Ontario, Canada produced by arthropods with at least 11 pairs of similar walking legs and a long tail-spine, presumably made by the extinct euthycarcinoids [103] (Figure 1E). Another site famous for its Cambrian traces of life on land is the middle to late Cambrian Blackberry Hill in central Wisconsin, which preserves diversity of arthropod trackways in a tidal flat and nearshore environment, along with the remains of the oldest euthycarcinoid, *Mosineia* [104]. Massive trackways from the intertidal zone left by euthycarcinoids with walking legs during the Cambrian and Ordovician indicate longer stays on land where these amphibious animals may have come in pursuit of shallow lagoons and freshwater pools [105], albeit their excursions on land may have been short-lived [106]. Other early arthropods to make temporary excursions to near-shore habitats were the trilobites, whose trace fossils in tidal-flat deposits are known since the Cambrian, albeit their traces were likely made subaqueally [107,108]. Trilobites possessed gill lamellae for respiration [109,110], which are unlikely to have provided them with the ability to survive on land for prolonged periods of time. Myriapods have been implicated in producing Ordovician backfilled burrows from Pennsylvania (445 Ma [111]), although the terrestrial nature of this deposit has been later disputed [36]. A slightly younger record of trackways and trails (*Diplichnites* and *Diplopodichnus*) from Cumbria, England (>450 Ma) records myriapods moving alongside the edges of ponds, but these were likely made under water [112,113]. Overall, locomotive traces documented throughout the Cambrian and Ordovician reinforce the view that aerial activities of arthropods (if not terrestrial arthropods) were common on the coasts and along the edges of freshwater bodies during this time. 

### 3.2. Body Fossil Evidence

The availability of land-dwelling arthropod body fossils is fundamentally constrained by the limited number of terrestrial formations before the Devonian and the limited interest these geological units have attracted in the past [42]. Fossils generally require a steady rate of sedimentation to preserve, which is not an easily achievable condition for minute soft-bodied arthropods inhabiting the soil or decaying vegetation matter. As such, palaeontologists have to rely on a restricted set of fossil localities that provide unusual preservation windows for their time.

The earliest relatives of myriapods in the fossil record are the Cambrian to Triassic euthycarcinoids, mentioned earlier for the terrestrial trace fossils. The affinities of this group have been traditionally difficult to pinpoint, but recent findings of exceptionally preserved Devonian specimens establish the group as the stem-group to myriapods [39]. These aquatic arthropods were amphibious, ranging from marine and brackish to freshwater deposits [114]. Their ventures on land have been variously interpreted as short migrations between ephemeral freshwater pools, grazing on microbial mats and detritus, or migrations to fertilise eggs on land like in modern horseshoe crabs [103,115]. Recent synchrotron studies revealed probable respiratory organs in a Devonian euthycarcinoid, consistent with an amphibious lifestyle [116]. Other early myriapod remains are known from the Silurian Kerrera (425 Ma) and Ludlow (420 Ma) deposits in the United Kingdom, albeit it is difficult to determine if they were truly terrestrial [59,117]. The earliest undoubtedly terrestrial fossil myriapod is the millipede *Pneumodesmus newmani* from Cowie in Scotland, originally regarded as Late Silurian [33], but more recently as Lower Devonian (414 Ma [67]). Its terrestrial ecology is indicated by the presence of spiracles.

The earliest hexapod fossils are the Early Devonian (~405 Ma) springtail *Rhyniella praecursor* [118,119] and the enigmatic *Leverhulmia mariae* [120], from the coeval Rhynie and Windyfield chert deposits in Scotland, which became preserved with extraordinary fidelity when silica-rich water from volcanic springs inundated hot springs and the surrounding land. While various systematic positions of the peculiar *Leverhulmia* have been proposed, *Rhyniella* is a crown-group springtail, not that different from species that inhabit soil and leaf litter today [121], suggesting that this clade of hexapods radiated well before the Early Devonian. Nonetheless, insect fossils before the Carboniferous are few; the Rhynie chert is followed by a window of 80 Ma (referred to as the ‘hexapod gap’) during which no insects are known [122]. The existence of pre-Devonian hexapods is a reasonable assumption, proposed already by early cladistic studies predating the molecular clock methodology [123]. Although a decade-old bounty of 1000 dollars has been put on an undisputable insect fossil from the pre-Devonian [124], this sum remains to be claimed. Instead, the hunt for early hexapods yielded a number of dubious records, like fossils only seen once and never again [125], suspected modern contaminants [106], and miss-identifications, such as purported Devonian insect wings that turned out to be malacostracan tail fans [126,127]. A recent review is provided by [128]. Others represent genuinely difficult fossils to interpret, such as the purported Devonian hexapod *Strudiella devonica* [129], which may however represent a decayed non-insect arthropod [130], or the Devonian *Wingertshellicus/Devonohexapodus* at once interpreted as an aquatic stem-hexapod [131], but not unequivocally accepted [132]. It is interesting to note that even in deposits such as the Rhynie chert where arthropod cuticles are not rare in some facies, the vast majority belong to arachnids, not hexapods as may be expected from modern ecosystems, where insects predominate. Winged insects only came to dominate terrestrial ecosystems by the Carboniferous, leading many to postulate that hexapods may have been species poor until the origin of with wings [42] that appear unequivocally in the fossil record in the latest Mississippian (~322 Ma [133]).

Among arachnids, we find the oldest fossil evidence of arthropod life on land, represented by scorpion remains from the Silurian (~437 Ma [134]). However, their terrestriality is not unambiguous due to the absence of bona fide terrestrial characters, such as book lungs, and have been found in aquatic or semi-aquatic deposits [135,136] (Figure 1H). Putative book lungs have been reported from a fossil scorpion from the Devonian Hunsrück Slate in Germany (~405 Ma [70]). Current molecular, phylogenomic and morphological evidence suggests, however, that scorpions are arachnids related to spiders [102,137,138], in a clade of mostly lung-bearing arachnids known as Arachnopulmonata. Within this clade, the latest phylogenomic results suggest that pseudoscorpions are the closest relatives of scorpions [66,74,139]. This phylogenetic position is hardly reconcilable with a marine origin of scorpions, suggesting that some of these ancestral scorpions may have secondarily returned to the aquatic environment, although without obvious marine adaptations. The earliest member of Trigonotarbida, a group of extinct terrestrial arachnids known to possess book lungs [140,141], is known from the Silurian (~420 Ma [63]) Ludford Lane in England [59]. Trigonotarbids persisted until the Permian and are known in stunning anatomic detail, in part thanks to their preservation in Rhynie chert [142]).

## 4. Reconciling Rocks and Clocks

### 4.1. Methodologies to Build Chronologies 

The abundant arthropod fossil record is informative on the diversity of the group, the historical evolution of morphological characters, and provides temporal guidelines for molecular dating. Solving the relative times of evolutionary divergences between species and clades in the geological past provides crucial information for dating the origin of terrestrial ecosystems. The reconstruction of these “timetrees”, or chronograms, is increasingly methodologically sophisticated and has become the backbone for comparative studies of evolutionary biology and palaeontology. Molecular data inform us both on the understanding of the tree’s branching pattern (the phylogeny) and, once calibrated with fossils, on the timing at which these branching events occurred (the timeline). The dates are inferred using the molecular clock technique [143], where the time elapsed since the divergence of different organisms or species is deduced from the differences between their DNA or amino acid sequences. To carry out these analyses, calibration points are routinely used where minimum ages are defined based on the oldest fossil evidence that can be unequivocally assigned to that node, that is, the origin of that group cannot be younger than its oldest fossil [144]. Node dating is the most widely used method [145], and it has developed a lot in recent years, with the implementation of Bayesian methods that allow assigning probabilities to age ranges and to other various parameters based on previous knowledge about the group in question [146]. While the chronologies constrain the real age of the lineages, the fossils inform us of when those organisms became numerically and ecologically abundant. Furthermore, including fossils in phylogenetic analyses helps arrive at more accurate trees and divergence time estimates [147,148,149]. Therefore, chronologies provide an essential conceptual framework for investigating the evolution of the first terrestrial ecosystems and the interactions over time between organisms and their environment.

### 4.2. Dating the Arthropod Terrestrialization

**Figure 4:**
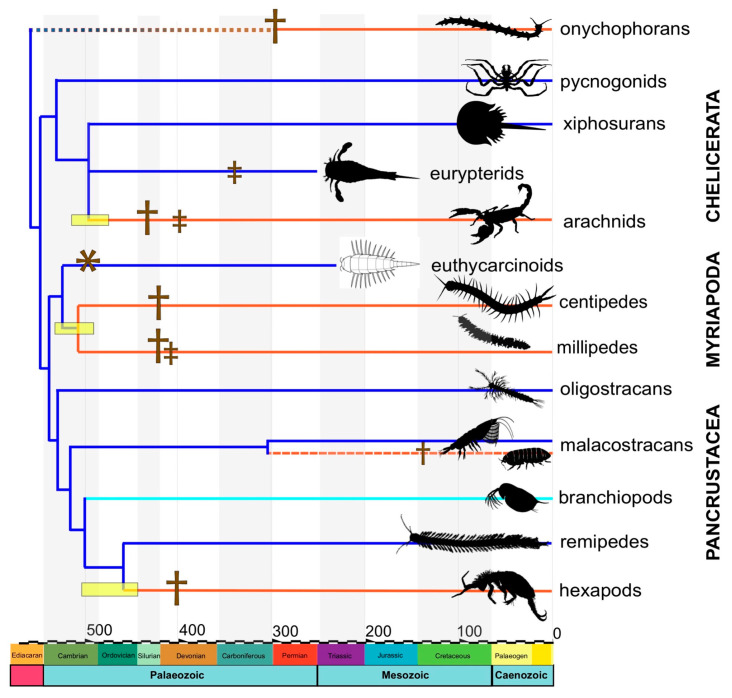
Schematic chronogram with divergence times between the most representative arthropod clades. The internal nodes of the tree fall into the mean estimated divergences taken from recent studies cited in the text. On the x-axis, time runs from most recent (right) to the past (left), and is expressed in millions of years. The yellow rectangles mark the credibility intervals for the different terrestrialization phenomena. The dagger symbol represents the oldest known fossil in that terrestrial group, the double dagger represents the oldest direct evidence of terrestrial breathing structures, and the asterisk the oldest trace evidence of terrestrial behaviour. The terrestrial groups are represented in orange colours while the marine clades in blue and turquoise for Branchiopoda (fresh water). Some of the silhouettes are from Phylopic (phylopic.org/; accessed on 5 November 2022).

Most recent chronologies of arthropod radiation (or subgroups of them) using molecular clocks are generally compatible with paleontological evidence, proposing an origin of the group between the end of the Ediacaran period and the beginning of the Cambrian (with credibility intervals falling with 95% of probability between 551–536 Ma) [37]. These studies also suggest the origin of arachnids and hexapods are in some consensus with the fossil evidence, preceding the oldest fossils by a few tens of thousands of years (Figure 4). In the case of chelicerates, the origin of terrestrial arachnids and of their main diversifications have been inferred to fall between the Cambrian and Ordovician (494–475 Ma) [66]. Molecular evolution rates were likely high during its origin, coinciding with a rapid cladogenesis [37]. When xiphosurans are nested within arachnids, the origin of this clade is inferred in ages comprising mostly Ediacaran [95] to Cambrian period [66]. For hexapods, the estimated ages vary in different studies between 520–450 Ma (summarised in [150]). Likewise, a Cambrian–Ordovician origin has been proposed for myriapods [25,78,83,151,152,153].

Consequently, there are certain differences when ages inferred from molecular dating studies are compared with the oldest fossil record, where arachnids first appeared in the Silurian (427 Ma) and hexapods in the Devonian (411 Ma). In the case of arachnids, it has been suggested that these differences may be due to the fact that the closest relative of arachnids is an extinct group. Eurypterids (also called ‘sea scorpions’) have been proposed as a possible sister group (Figure 1G). These aquatic organisms emerged during the Ordovician (~467 Ma) and represented an important component of marine fauna until they disappeared from the fossil record during the end-Permian mass extinction (~252 Ma) [10]. It seems that they could make inroads into the terrestrial environment, as suggested by ichnofossils, and recent studies show that they had respiratory structures adapted to breathing air, possibly since the Cambrian–Ordovician [40]. The latter study suggests that their ancestor may have been semi-terrestrial, similar to eurypterids. Regarding the origin of myriapods, the divergence times inferred are substantially older (524–505 Ma) than their oldest fossil evidence, and they firmly place the earliest members of this group in the Cambrian [37,78,151,152,153], despite the fact that its oldest fossil is 414 Ma (Figure 4). The reinterpretation of Euthycarcinoidea as the closest relative of myriapods based on the similarity of mouth and eye structures bridges this gap between the fossil record and molecular clocks [39].

### 4.3. Reconciling the Fossil and Molecular Evidence

The discrepancies between the results derived from molecular clocks and the oldest fossil evidence may be related to the nature of the rock record, especially to the rarity of terrestrial sediments from the Cambrian to the Silurian. It has been suggested that Euramerica, the region from which much of the data on the first terrestrial arthropods and plant megafossils are derived, is almost absent from terrestrial sediments before the upper Silurian and that these are not more widespread until the Early Devonian [50]. This temporal bias in the rock record possibly affects the fossil record of terrestrial organisms and may explain part of the mismatch between molecular and fossil dates. The discrepancy may also be explained by failures in the molecular clock methodology, particularly with the node dating strategy. A recent method has been developed to estimate divergence time in a total-evidence framework, where fossils are directly integrated into the combined analysis of molecular data from living species with morphological data from fossils and living groups [154]. In the process of reconstructing kinship relationships and dating them, fossils are incorporated without having to determine their phylogenetic position a priori, and therefore this phylogenetic uncertainty can be directly integrated in the analysis. Some studies suggest that this approach improves divergence time estimates [147]. Computational limitations currently limit the application of this methodology to determine deep divergences. However, the field is advancing rapidly, and it is predicted that soon these methodologies will help to establish the affinity of fossils, and more carefully assign the age of the lineages and the different terrestrialization processes [155].

## 5. Conclusions

Ephemeral terrestrial habitats have existed for at least 1 billion years. However, animal terrestrialization and the consequent formation of more complex habitats has been a much more recent process. How recent remains a point of contention. The fossil record provides the only direct source of data to understand the temporal acquisition of characters, while phylogenies and molecular clocks complement this record to constrain the timing of the origin of these groups. The most recent molecular dating suggests that land plants were already present in the middle Cambrian to Early Ordovician [38], although other recent molecular clock estimates push this date back even further, into the Precambrian [156]. Similarly, recent molecular dating studies also suggest a concomitant colonization of the land by arthropods. If myriapods and arachnids really colonized the terrestrial environment so early, it would be possible that millipedes, a group of detritivore myriapods, fed on bacterial mats on the shoreline. Arachnids are a predominantly predatory group, suggesting that they must have originated from a diverse ecosystem. In this scenario, arachnids could have myriapods as potential prey. These ecologies represent habitats highly unfavourable to fossilization, such as high-energy environments characterized by erosion rather than deposition [157]. It is not surprising, then, that direct palaeontological insights may be limited in these cases, and molecular inference can step in to fill the gap. 

## Data Availability

Not applicable.

## References

[1] Betts H.C., Puttick M.N., Clark J.W., Williams T.A., Donoghue P.C.J., Pisani D. (2018). Integrated Genomic and Fossil Evidence Illuminates Life’s Early Evolution and Eukaryote Origin. Nat. Ecol. Evol..

[2] Schopf T.J.M. (1984). Rates of Evolution and the Notion of “Living Fossils”. Annu. Rev. Earth Planet. Sci..

[3] Allwood A.C., Rosing M.T., Flannery D.T., Hurowitz J.A., Heirwegh C.M. (2018). Reassessing Evidence of Life in 3700-Million-Year-Old Rocks of Greenland. Nature.

[4] Cavalazzi B., Lemelle L., Simionovici A., Cady S.L., Russell M.J., Bailo E., Canteri R., Enrico E., Manceau A., Maris A. (2021). Cellular Remains in a ~3.42-Billion-Year-Old Subseafloor Hydrothermal Environment. Sci. Adv..

[5] Sogin M.L. (1994). The origin of eukaryotes and evolution into Major Kingdoms. Early Life on Earth.

[6] Zhu S., Zhu M., Knoll A.H., Yin Z., Zhao F., Sun S., Qu Y., Shi M., Liu H. (2016). Decimetre-Scale Multicellular Eukaryotes from the 1.56-Billion-Year-Old Gaoyuzhuang Formation in North China. Nat. Commun..

[7] Paterson S., Vogwill T., Buckling A., Benmayor R., Spiers A.J., Thomson N.R., Quail M., Smith F., Walker D., Libberton B. (2010). Antagonistic Coevolution Accelerates Molecular Evolution. Nature.

[8] Erwin D.H. (2011). Evolutionary Uniformitarianism. Dev. Biol..

[9] Erwin D.H., Valentine J.W. (2013). The Cambrian Explosion: The Construction of Animal Biodiversity.

[10] Gaines R.R. (2014). Burgess Shale-Type Preservation and Its Distribution in Space and Time. Paleontol. Soc. Pap..

[11] Yang C., Li X.-H., Zhu M., Condon D.J., Chen J. (2018). Geochronological Constraint on the Cambrian Chengjiang Biota, South China. J. Geol. Soc..

[12] Littlewood D.T.J., Rohde K.K. (2005). Marine parasites and the tree of life. Marine Parasitology.

[13] Selden P.A., Jeram A.J. (1989). Palaeophysiology of Terrestrialisation in the Chelicerata. Earth Environ. Sci. Trans. R. Soc. Edinb..

[14] De Baets K., Dentzien-Dias P., Harrison G.W.M., Littlewood D.T.J., Parry L.A., De Baets K., Huntley J.W. (2021). Fossil Constraints on the Timescale of Parasitic Helminth Evolution. The Evolution and Fossil Record of Parasitism: Identification and Macroevolution of Parasites.

[15] Muscente A.D., Schiffbauer J.D., Broce J., Laflamme M., O’Donnell K., Boag T.H., Meyer M., Hawkins A.D., Huntley J.W., McNamara M. (2017). Exceptionally Preserved Fossil Assemblages through Geologic Time and Space. Gondwana Res..

[16] Vermeij G.J., Watson-Zink V.M. (2022). Terrestrialization in Gastropods: Lineages, Ecological Constraints and Comparisons with Other Animals. Biol. J. Linn. Soc..

[17] Garwood R.J., Edgecombe G.D., Charbonnier S., Chabard D., Sotty D., Giribet G. (2016). Carboniferous Onychophora from Montceau-Les-Mines, France, and Onychophoran Terrestrialization. Invert. Biol..

[18] van Straalen N.M. (2021). Evolutionary Terrestrialization Scenarios for Soil Invertebrates. Pedobiologia.

[19] Holterman M., Schratzberger M., Helder J. (2019). Nematodes as Evolutionary Commuters between Marine, Freshwater and Terrestrial Habitats. Biol. J. Linn. Soc..

[20] Okamura B., Gruhl A., De Baets K. (2022). Evolutionary Transitions of Parasites between Freshwater and Marine Environments. Integr. Comp. Biol..

[21] Tchesunov A.V., Ivanenko V.N. (2022). What Is the Difference between Marine and Limnetic-Terrestrial Associations of Nematodes with Invertebrates?. Integr. Zool..

[22] Guidetti R., Bertolani R., Schill R.O. (2018). Paleontology and Molecular Dating. Water Bears: The Biology of Tardigrades.

[23] Sluys R. (2019). The Evolutionary Terrestrialization of Planarian Flatworms (Platyhelminthes, Tricladida, Geoplanidae): A Review and Research Programme. Zoosyst. Evol..

[24] Dunlop J.A., Scholtz G., Selden P.A., Minelli A., Boxshall G., Fusco G. (2013). Water-to-Land Transitions. Arthropod Biology and Evolution: Molecules, Development, Morphology.

[25] Lozano-Fernandez J., Carton R., Tanner A.R., Puttick M.N., Blaxter M., Vinther J., Olesen J., Giribet G., Edgecombe G.D., Pisani D. (2016). A Molecular Palaeobiological Exploration of Arthropod Terrestrialization. Phil. Trans. Roy. Soc. B..

[26] Felsenstein J. (1985). Phylogenies and the Comparative Method. Am. Nat..

[27] Benton M.J. (2010). The Origins of Modern Biodiversity on Land. Phil. Trans. R. Soc. B.

[28] Román-Palacios C., Moraga-López D., Wiens J.J. (2022). The Origins of Global Biodiversity on Land, Sea and Freshwater. Ecol. Lett..

[29] Clarke J.T., Warnock R.C.M., Donoghue P.C.J. (2011). Establishing a Time-Scale for Plant Evolution. New Phytol..

[30] Prave A.R. (2002). Life on Land in the Proterozoic: Evidence from the Torridonian Rocks of Northwest Scotland. Geology.

[31] Buatois L.A., Davies N.S., Gibling M.R., Krapovickas V., Labandeira C.C., MacNaughton R.B., Mángano M.G., Minter N.J., Shillito A.P. (2022). The Invasion of the Land in Deep Time: Integrating Paleozoic Records of Paleobiology, Ichnology, Sedimentology, and Geomorphology. Integr. Comp. Biol..

[32] Xu H., Wang K., Huang Z., Tang P., Wang Y., Liu B., Yan W. (2022). The Earliest Vascular Land Plants from the Upper Ordovician of China 2022. Biol. Sci..

[33] Wilson H.M., Anderson L.I. (2004). Morphology and Taxonomy of Paleozoic Millipedes (Diplopoda: Chilognatha: Archipolypoda) from Scotland. J. Paleontol..

[34] Dunlop J. (1996). A Trigonotarbid Arachnid from the Upper Silurian of Shropshire. Palaeontology.

[35] Ward P., Labandeira C., Laurin M., Berner R.A. (2006). Confirmation of Romer’s Gap as a Low Oxygen Interval Constraining the Timing of Initial Arthropod and Vertebrate Terrestrialization. Proc. Natl. Acad. Sci. USA.

[36] Davies N.S., Rygel M.C., Gibling M.R. (2010). Marine Influence in the Upper Ordovician Juniata Formation (Potters Mills, Pennsylvania): Implications for the History of Life on Land. PALAIOS.

[37] Lozano-Fernandez J., Tanner A.R., Puttick M.N., Vinther J., Edgecombe G.D., Pisani D. (2020). A Cambrian–Ordovician Terrestrialization of Arachnids. Front. Genet..

[38] Morris J.L., Puttick M.N., Clark J.W., Edwards D., Kenrick P., Pressel S., Wellman C.H., Yang Z., Schneider H., Donoghue P.C. (2018). The Timescale of Early Land Plant Evolution. Proc. Natl. Acad. Sci. USA.

[39] Edgecombe G.D., Strullu-Derrien C., Góral T., Hetherington A.J., Thompson C., Koch M. (2020). Aquatic Stem Group Myriapods Close a Gap between Molecular Divergence Dates and the Terrestrial Fossil Record. Proc. Natl. Acad. Sci. USA.

[40] Lamsdell J.C., McCoy V.E., Perron-Feller O.A., Hopkins M.J. (2020). Air Breathing in an Exceptionally Preserved 340-Million-Year-Old Sea Scorpion. Curr. Biol..

[41] Royer D.L., Donnadieu Y., Park J., Kowalczyk J., Goddéris Y. (2014). Error Analysis of CO_2_ and O_2_ Estimates from the Long-Term Geochemical Model GEOCARBSULF. Am. J. Sci..

[42] Schachat S.R., Labandeira C.C., Saltzman M.R., Cramer B.D., Payne J.L., Boyce C.K. (2018). Phanerozoic p O_2_ and the Early Evolution of Terrestrial Animals. Proc. R. Soc. Lond. B.

[43] Gregor B. (1970). Denudation of the Continents. Nature.

[44] Blatt H., Jones R.L. (1975). Proportions of Exposed Igneous, Metamorphic, and Sedimentary Rocks. GSA Bull..

[45] Khain V.Y., Ronov A.B., Seslavinskiy K.B. (1978). Silurian Lithologic Associations of the World. Int. Geol. Rev..

[46] Ronov A.B. (1982). The Earth’s Sedimentary Shell (Quantitative Patterns of Its Structure, Compositions, and Evolution). Int. Geol. Rev..

[47] Ronov A.B., Khain V.E., Balukhovsky A.N., Seslavinsky K.B. (1980). Quantitative Analysis of Phanerozoic Sedimentation. Sed. Geol..

[48] Smith A.B., McGowan A.J. (2007). The Shape of the Phanerozoic Marine Palaeodiversity Curve: How Much Can Be Predicted from the Sedimentary Rock Record of Western Europe?. Palaeontology.

[49] Robardet M., Blaise J., Bouyx E., Gourvennec R., Lardeux H., Le Hérissé A., Le Menn J., Melou M., Paris F., Plusquellec Y. (1993). Palaeogeography of Western Europe from the Ordovician to the Devonian. Bull. Soc. Geol. Fr..

[50] Kenrick P., Wellman C.H., Schneider H., Edgecombe G.D. (2012). A Timeline for Terrestrialization: Consequences for the Carbon Cycle in the Palaeozoic. Phil. Trans. R. Soc. B.

[51] Zhang Z.-Q. (2013). Animal Biodiversity: An Outline of Higher-Level Classification and Survey of Taxonomic Richness (Addenda 2013). Zootaxa.

[52] Maloof A.C., Rose C.V., Beach R., Samuels B.M., Calmet C.C., Erwin D.H., Poirier G.R., Yao N., Simons F.J. (2010). Possible Animal-Body Fossils in Pre-Marinoan Limestones from South Australia. Nat. Geosci..

[53] Wolfe J.M., Daley A.C., Legg D.A., Edgecombe G.D. (2016). Fossil Calibrations for the Arthropod Tree of Life. Earth Sci. Rev..

[54] Edgecombe G.D. (2010). Arthropod Phylogeny: An Overview from the Perspectives of Morphology, Molecular Data and the Fossil Record. Arthropod Struct. Dev..

[55] Grimaldi D., Engel M.S. (2005). Evolution of the Insects.

[56] Govorushko S. (2019). Economic and Ecological Importance of Termites: A Global Review. Entomol. Sci..

[57] Brusca R.C., Brusca G.J. (2003). Invertebrates.

[58] Little C. (1983). The Colonisation of Land: Origins and Adaptations of Terrestrial Animals.

[59] Jeram A.J., Selden P.A., Edwards D. (1990). Land Animals in the Silurian: Arachnids and Myriapods from Shropshire, England. Science.

[60] Shear W., Selden P. (1995). Eoarthropleura (Arthropoda, Arthropleurida) from the Silurian of Britain and the Devonian of North America. Neues Jahrb. Geol. Palaontol. Abh..

[61] Shear W.A., Jeram A.J., Selden P. (1998). Centiped Legs (Arthropoda, Chilopoda, Scutigeromorpha) from the Silurian and Devonian of Britain and the Devonian of North America. Am. Mus. Novit..

[62] Dunlop J.A. (1999). A Replacement Name for the Trigonotarbid Arachnid *Eotarbus* Dunlop. Palaeontology.

[63] Brookfield M.E., Catlos E.J., Suarez S.E. (2020). Myriapod Divergence Times Differ between Molecular Clock and Fossil Evidence: U/Pb Zircon Ages of the Earliest Fossil Millipede-Bearing Sediments and Their Significance. Hist. Biol..

[64] Marshall J.E.A. (1991). Palynology of the Stonehaven Group, Scotland: Evidence for a Mid Silurian Age and Its Geological Implications. Geol. Mag..

[65] Wellman C.H. (1993). A Land Plant Microfossil Assemblage of Mid Silurian Age from the Stonehaven Group, Scotland. J. Micropalaeontol..

[66] Howard R.J., Puttick M.N., Edgecombe G.D., Lozano-Fernandez J. (2020). Arachnid Monophyly: Morphological, Palaeontological and Molecular Support for a Single Terrestrialization within Chelicerata. Arthropod Struct. Devel..

[67] Suarez S.E., Brookfield M.E., Catlos E.J., Stöckli D.F. (2017). A U-Pb Zircon Age Constraint on the Oldest-Recorded Air-Breathing Land Animal. PLoS ONE.

[68] Kutscher F. (1971). Friedrich Beiträge Zur Sedimentation Und Fossilführung Des Hunsrückschiefers 32. *Palaeoscorpius devonicus*, Ein Devonischer Skorpion. Jahrb. Nassau. Ver. Naturkd..

[69] Lehmann W.M. (1944). *Palaeoscorpius devonicus* Ng, n. Sp., Ein Skorpion Aus Dem Rheinischen Unterdevon. N. Jahrb. Geol. Palaontol. Monat..

[70] Kühl G., Bergmann A., Dunlop J., Garwood R.J., Rust J. (2012). Redescription and Palaeobiology of *Palaeoscorpius devonicus* Lehmann, 1944 from the Lower Devonian Hunsrück Slate of Germany. Palaeontology.

[71] Zrzavý J., Hypša V., Vlášková M., Fortey R.A., Thomas R.H. (1998). Arthropod Phylogeny: Taxonomic Congruence, Total Evidence and Conditional Combination Approaches to Morphological and Molecular Data Sets. Arthropod Relationships.

[72] Giribet G., Edgecombe G.D. (2019). The Phylogeny and Evolutionary History of Arthropods. Curr. Biol..

[73] Legg D.A., Sutton M.D., Edgecombe G.D. (2013). Arthropod Fossil Data Increase Congruence of Morphological and Molecular Phylogenies. Nat. Commun..

[74] Ballesteros J.A., Santibáñez-López C.E., Baker C.M., Benavides L.R., Cunha T.J., Gainett G., Ontano A.Z., Setton E.V.W., Arango C.P., Gavish-Regev E. (2022). Comprehensive Species Sampling and Sophisticated Algorithmic Approaches Refute the Monophyly of Arachnida. Mol. Biol. Evol..

[75] Tihelka E., Cai C., Giacomelli M., Lozano-Fernandez J., Rota-Stabelli O., Huang D., Engel M.S., Donoghue P.C.J., Pisani D. (2021). The Evolution of Insect Biodiversity. Curr. Biol..

[76] Bäcker H., Fanenbruck M., Wägele J.W. (2008). A Forgotten Homology Supporting the Monophyly of Tracheata: The Subcoxa of Insects and Myriapods Re-Visited. Zool. Anz. J. Comp. Zool..

[77] Giribet G., Edgecombe G.D. (2012). Reevaluating the Arthropod Tree of Life. Annu. Rev. Entomol..

[78] Fernández R., Edgecombe G.D., Giribet G., Edgecombe G.D., Giribet G. (2018). Phylogenomics Illuminates the Backbone of the Myriapoda Tree of Life and Reconciles Morphological and Molecular Phylogenies. Sci. Rep..

[79] Rota-Stabelli O., Campbell L., Brinkmann H., Edgecombe G.D., Longhorn S.J., Peterson K.J., Pisani D., Philippe H., Telford M.J. (2011). A Congruent Solution to Arthropod Phylogeny: Phylogenomics, MicroRNAs and Morphology Support Monophyletic Mandibulata. Proc. R. Soc. B.

[80] Fernández R., Edgecombe G.D., Giribet G. (2016). Exploring Phylogenetic Relationships within Myriapoda and the Effects of Matrix Composition and Occupancy on Phylogenomic Reconstruction. Syst. Biol..

[81] Szucsich N.U., Bartel D., Blanke A., Böhm A., Donath A., Fukui M., Grove S., Liu S., Macek O., Machida R. (2020). Four Myriapod Relatives—But Who Are Sisters? No End to Debates on Relationships among the Four Major Myriapod Subgroups. BMC Evol. Biol..

[82] Wang J., Bai Y., Zhao H., Mu R., Dong Y. (2021). Reinvestigating the Phylogeny of Myriapoda with More Extensive Taxon Sampling and Novel Genetic Perspective. PeerJ.

[83] Benavides L.R., Edgecombe G.D., Giribet G. (2022). Re-Evaluating and Dating Myriapod Diversification with Phylotranscriptomics under a Regime of Dense Taxon Sampling. Mol. Phylogenetics Evol..

[84] Schwentner M., Combosch D.J., Nelson J.P., Giribet G. (2017). A Phylogenomic Solution to the Origin of Insects by Resolving Crustacean-Hexapod Relationships. Curr. Biol..

[85] Regier J.C., Shultz J.W., Zwick A., Hussey A., Ball B., Wetzer R., Martin J.W., Cunningham C.W., Shultz J.W., Zwick A. (2010). Arthropod Relationships Revealed by Phylogenomic Analysis of Nuclear Protein-Coding Sequences. Nature.

[86] Lozano-Fernandez J., Giacomelli M., Fleming J.F., Chen A., Vinther J., Thomsen P.F., Glenner H., Palero F., Legg D.A., Iliffe T.M. (2019). Pancrustacean Evolution Illuminated by Taxon-Rich Genomic-Scale Data Sets with an Expanded Remipede Sampling. Genome Biol. Evol..

[87] Yager J. (1981). Remipedia, a New Class of Crustacea from a Marine Cave in the Bahamas. J. Crustacean Biol..

[88] Schmalfuss H. (2003). World Catalog of Terrestrial Isopods (Isopoda: Oniscidea). Stuttg. Beitr. Naturkd. A.

[89] Richardson A., Araujo P.B. (2015). Lifestyles of Terrestrial Crustacean. The Natural History of the Crustacea. Lifestyles and Feeding Biology.

[90] Broly P., Deville P., Maillet S. (2013). The Origin of Terrestrial Isopods (Crustacea: Isopoda: Oniscidea). Evol. Ecol..

[91] Elisabeth H. (2011). Evolutionary Adaptation of Oniscidean Isopods to Terrestrial Life: Structure, Physiology and Behavior. Terr. Arthropod Rev..

[92] Schmidt C. (2008). Phylogeny of the Terrestrial Isopoda (Oniscidea): A Review. Arthr. Syst. Phyl..

[93] Dimitriou A.C., Taiti S., Sfenthourakis S. (2019). Genetic Evidence against Monophyly of Oniscidea Implies a Need to Revise Scenarios for the Origin of Terrestrial Isopods. Sci. Rep..

[94] Tabacaru I., Giurginca A. (2021). The Monophyly and the Classification of the Terrestrial Isopods (Crustacea, Isopoda, Oniscidea). Trav. Inst. Speol. Emile Racovitza.

[95] Ballesteros J.A., Sharma P.P. (2019). A Critical Appraisal of the Placement of Xiphosura (Chelicerata) with Account of Known Sources of Phylogenetic Error. Syst. Biol..

[96] Shultz J.W. (2007). A Phylogenetic Analysis of the Arachnid Orders Based on Morphological Characters. Zool. J. Linn. Soc..

[97] Shultz J.W. (1990). Evolutionary Morphology and Phylogeny of Arachnida. Cladistics.

[98] Bicknell R.D.C., Pates S. (2020). Pictorial Atlas of Fossil and Extant Horseshoe Crabs, with Focus on Xiphosurida. Front. Earth Sci..

[99] Gould S.J. (1970). Dollo on Dollo’s Law: Irreversibility and the Status of Evolutionary Laws. J. Hist. Biol..

[100] Lamsdell J.C. (2019). Evolutionary History of the Dynamic Horseshoe Crab. Int. Wader Stud..

[101] Bicknell R.D.C., Kimmig J., Budd G.E., Legg D.A., Bader K.S., Haug C., Kaiser D., Laibl L., Tashman J.N., Campione N.E. (2022). Habitat and Developmental Constraints Drove 330 Million Years of Horseshoe Crab Evolution. Biol. J. Linn. Soc..

[102] Lozano-Fernandez J., Tanner A.R., Giacomelli M., Carton R., Vinther J., Edgecombe G.D., Pisani D. (2019). Increasing Species Sampling in Chelicerate Genomic-Scale Datasets Provides Support for Monophyly of Acari and Arachnida. Nat. Commun..

[103] MacNaughton R.B., Cole J.M., Dalrymple R.W., Braddy S.J., Briggs D.E.G., Lukie T.D. (2002). First Steps on Land: Arthropod Trackways in Cambrian-Ordovician Eolian Sandstone, Southeastern Ontario, Canada. Geology.

[104] Collette J.H., Hagadorn J.W. (2010). Three-Dimensionally Preserved Arthropods from Cambrian Lagerstätten of Quebec and Wisconsin. J. Paleontol..

[105] Vaccari N.E., Edgecombe G.D., Escudero C., Edgecombe G.D., Escudero C. (2004). Cambrian Origins and Affinities of an Enigmatic Fossil Group of Arthropods. Nature.

[106] Braddy S.J., Gass K.C., Gass T.C. (2022). Fossils of Blackberry Hill, Wisconsin, USA: The First Animals on Land, 500 Million Years Ago. Geol. Today.

[107] Mángano M.G., Buatois L.A., Astini R., Rindsberg A.K. (2014). Trilobites in Early Cambrian Tidal Flats and the Landward Expansion of the Cambrian Explosion. Geology.

[108] Mángano M.G., Buatois L.A., Waisfeld B.G., Muñoz D.F., Vaccari N.E., Astini R.A. (2021). Were All Trilobites Fully Marine? Trilobite Expansion into Brackish Water during the Early Palaeozoic. Proc. R. Soc. B.

[109] Suzuki Y., Bergström J. (2008). Respiration in Trilobites: A Reevaluation. GFF.

[110] Hou J., Hughes N.C., Hopkins M.J. (2021). The Trilobite Upper Limb Branch Is a Well-Developed Gill. Sci. Adv..

[111] Retallack G.J. (2001). *Scoyenia* Burrows from Ordovician Palaeosols of the Juniata Formation in Pennsylvania. Palaeontology.

[112] Shillito A.P., Davies N.S. (2018). Death near the Shoreline, Not Life on Land: Ordovician Arthropod Trackways in the Borrowdale Volcanic Group, UK. Geology.

[113] Johnson E.W., Briggs D.E.G., Suthren R.J., Wright J.L., Tunnicliff S.P. (1994). Non-Marine Arthropod Traces from the Subaerial Ordovician Borrowdale Volcanic Group, English Lake District. Geol. Mag..

[114] Ortega-Hernández J., Legg D.A., Tremewan J., Braddy S.J. (2010). Euthycarcinoids. Geol. Today.

[115] Collette J.H., Gass K.G., Hagadorn J.W. (2012). *Protichnites eremita* Unshelled? Experimental Model-Based Neoichnology and New Evidence for a Euthycarcinoid Affinity for This Ichnospecies. J. Paleontol..

[116] Gueriau P., Lamsdell J.C., Wogelius R.A., Manning P.L., Egerton V.M., Bergmann U., Bertrand L., Denayer J. (2020). A New Devonian Euthycarcinoid Reveals the Use of Different Respiratory Strategies during the Marine-to-Terrestrial Transition in the Myriapod Lineage. R. Soc. Open Sci..

[117] Trewin N.H., Gurr P.R., Jones R.B., Gavin P. (2012). The Biota, Depositional Environment and Age of the Old Red Sandstone of the Island of Kerrera, Scotland. Scott. J. Geol..

[118] Scourfield D.J. (1940). The Oldest Known Fossil Insect. Nature.

[119] Whalley P., Jarzembowski E.A. (1981). A New Assessment of *Rhyniella*, the Earliest Known Insect, from the Devonian of Rhynie, Scotland. Nature.

[120] Fayers S.R., Trewin N.H. (2005). A Hexapod from the Early Devonian Windyfield Chert, Rhynie, Scotland. Palaeontology.

[121] Greenslade P., Whalley P.E.S. The Systematic Position of *Rhyniella praecursor* (Hirst and Malik, the Earliest Known Hexapod. Proceedings of the 2nd International Seminar on Apterygota.

[122] Shear W.A. (2012). An Insect to Fill the Gap. Nature.

[123] Kukalová-Peck J. (1987). New Carboniferous Diplura, Monura, and Thysanura, the Hexapod Ground Plan, and the Role of Thoracic Side Lobes in the Origin of Wings (Insecta). Can. J. Zool..

[124] Hošek P. (1994). Fossil Insect Diversity. Vesmír.

[125] Crowson R.A. (1985). Comments on Insecta of the Rhynie Chert. Entomol. Gener..

[126] Rohdendorf B.B. (1972). Devonskie Eopteridy-Ne Nasekomye a Rakoobraznye Eumalacostraca. Entomol. Oboz..

[127] Schram F.R. (1980). Miscellaneous Late Paleozoic Malacostraca of the Soviet Union. J. Paleontol..

[128] Haug C., Haug J.T. (2017). The Presumed Oldest Flying Insect: More Likely a Myriapod?. PeerJ.

[129] Garrouste R., Clément G., Nel P., Engel M.S., Grandcolas P., D’Haese C., Lagebro L., Denayer J., Gueriau P., Lafaite P. (2012). A Complete Insect from the Late Devonian Period. Nature.

[130] Hörnschemeyer T., Haug J.T., Bethoux O., Beutel R.G., Charbonnier S., Hegna T.A., Koch M., Rust J., Wedmann S., Bradler S. (2013). Is *Strudiella* a Devonian Insect?. Nature.

[131] Haas F., Waloszek D., Hartenberger R. (2003). *Devonohexapodus bocksbergensis*, a New Marine Hexapod from the Lower Devonian Hunsrück Slates, and the Origin of Atelocerata and Hexapoda. Org. Divers. Evol..

[132] Kühl G., Rust J. (2009). *Devonohexapodus Bocksbergensis* Is a Synonym of *Wingertshellicus backesi* (Euarthropoda)—No Evidence for Marine Hexapods Living in the Devonian Hunsrück Sea. Org. Divers. Evol..

[133] Brauckmann C., Brauckmann B., Groning E. (1994). The Stratigraphical Position of the Oldest Known Pterygota (Insecta. Carboniferous, Namurian). Ann. Soc. Géol. Belg..

[134] Dunlop J.A., Erik Tetlie O., Prendini L. (2008). Reinterpretation of the Silurian Scorpion *Proscorpius osborni* (Whitfield): Integrating Data from Palaeozoic and Recent Scorpioans. Palaeontology.

[135] Waddington J., Rudkin D.M., Dunlop J.A. (2015). A New Mid-Silurian Aquatic Scorpion—One Step Closer to Land?. Biol. Lett..

[136] Wendruff A.J., Babcock L.E., Wirkner C.S., Kluessendorf J., Mikulic D.G. (2020). A Silurian Ancestral Scorpion with Fossilised Internal Anatomy Illustrating a Pathway to Arachnid Terrestrialisation. Sci. Rep..

[137] Sharma P.P., Kaluziak S.T., Pérez-Porro A.R., González V.L., Hormiga G., Wheeler W.C., Giribet G. (2014). Phylogenomic Interrogation of Arachnida Reveals Systemic Conflicts in Phylogenetic Signal. Mol. Biol. Evol..

[138] Leite D.J., Baudouin-Gonzalez L., Iwasaki-Yokozawa S., Lozano-Fernandez J., Turetzek N., Akiyama-Oda Y., Prpic N.-M., Pisani D., Oda H., Sharma P.P. (2018). Homeobox Gene Duplication and Divergence in Arachnids. Mol. Biol. Evol..

[139] Ontano A.Z., Gainett G., Aharon S., Ballesteros J.A., Benavides L.R., Corbett K.F., Gavish-Regev E., Harvey M.S., Monsma S., Santibáñez-López C.E. (2021). Taxonomic Sampling and Rare Genomic Changes Overcome Long-Branch Attraction in the Phylogenetic Placement of Pseudoscorpions. Mol. Biol. Evol..

[140] Claridge M.F., Lyon A.G. (1961). Lung-Books in the Devonian Palæocharinidae (Arachnida). Nature.

[141] Kamenz C., Dunlop J.A., Scholtz G., Kerp H., Hass H. (2008). Microanatomy of Early Devonian Book Lungs. Biol. Lett..

[142] Dunlop J.A., Garwood R.J. (2018). Terrestrial Invertebrates in the Rhynie Chert Ecosystem. Phil. Trans. R. Soc. B.

[143] Zuckerkandl E., Pauling L., Bryson V., Vogel H.J. (1965). Evolutionary Divergence and Convergence in Proteins. Evolving Genes and Proteins.

[144] Parham J.F., Donoghue P.C., Bell C.J., Calway T.D., Head J.J., Holroyd P.A., Inoue J.G., Irmis R.B., Joyce W.G., Ksepka D.T. (2011). Best Practices for Justifying Fossil Calibrations. Syst. Biol..

[145] Yang Z., Rannala B. (2006). Bayesian Estimation of Species Divergence Times under a Molecular Clock Using Multiple Fossil Calibrations with Soft Bounds. Mol. Biol. Evol..

[146] dos Reis M., Thawornwattana Y., Angelis K., Telford M.J., Donoghue P.C.J., Yang Z. (2015). Uncertainty in the Timing of Origin of Animals and the Limits of Precision in Molecular Timescales. Curr. Biol..

[147] Mongiardino Koch N., Parry L.A. (2020). Death Is on Our Side: Paleontological Data Drastically Modify Phylogenetic Hypotheses. Syst. Biol..

[148] Mongiardino Koch N., Garwood R.J., Parry L.A. (2022). Inaccurate Fossil Placement Does Not Compromise Tip-Dated Divergence Times. bioRxiv.

[149] Wright A.M., Bapst D.W., Barido-Sottani J., Warnock R.C.M. (2022). Integrating Fossil Observations into Phylogenetics Using the Fossilized Birth–Death Model. Ann. Rev. Earth Planet. Sci..

[150] Klopfstein S. (2021). The Age of Insects and the Revival of the Minimum Age Tree. Austral. Entomol..

[151] Rota-Stabelli O., Lartillot N., Philippe H., Pisani D. (2013). Serine Codon-Usage Bias in Deep Phylogenomics: Pancrustacean Relationships as a Case Study. Syst. Biol..

[152] Rehm P., Meusemann K., Borner J., Misof B., Burmester T. (2014). Phylogenetic Position of Myriapoda Revealed by 454 Transcriptome Sequencing. Mol. Phylogenet. Evol..

[153] Miyazawa H., Ueda C., Yahata K., Su Z.-H., Ueda C., Yahata K., Su Z.-H. (2014). Molecular Phylogeny of Myriapoda Provides Insights into Evolutionary Patterns of the Mode in Post-Embryonic Development. Sci. Rep..

[154] Ronquist F., Klopfstein S., Vilhelmsen L., Schulmeister S., Murray D.L., Rasnitsyn A.P. (2012). A Total-Evidence Approach to Dating with Fossils, Applied to the Early Radiation of the Hymenoptera. Syst. Biol..

[155] Howard R.J., Edgecombe G.D., Legg D.A., Pisani D., Lozano-Fernandez J. (2019). Exploring the Evolution and Terrestrialization of Scorpions (Arachnida: Scorpiones) with Rocks and Clocks. Org. Divers. Evol..

[156] Su D., Yang L., Shi X., Ma X., Zhou X., Hedges S.B., Zhong B. (2021). Large-Scale Phylogenomic Analyses Reveal the Monophyly of Bryophytes and Neoproterozoic Origin of Land Plants. Mol. Biol. Evol..

[157] Parry L.A., Smithwick F., Nordén K.K., Saitta E.T., Lozano-Fernandez J., Tanner A.R., Caron J.-B., Edgecombe G.D., Briggs D.E.G., Vinther J. (2018). Soft-Bodied Fossils Are Not Simply Rotten Carcasses—Toward a Holistic Understanding of Exceptional Fossil Preservation. BioEssays.

